# Supplementary material for: Modulation of Phosphate Deficiency-Induced Metabolic Changes by Iron Availability in Arabidopsis thaliana
Source: Int J Mol Sci. 2021 Jul 16;22(14):7609. doi: 10.3390/ijms22147609 (PMC8306678; doi:10.3390/ijms22147609)
Supplement: Supplementary file 1 [file ijms-22-07609-s001.zip › Chutia-etal-Figure S1.pptx]

## Slide 1
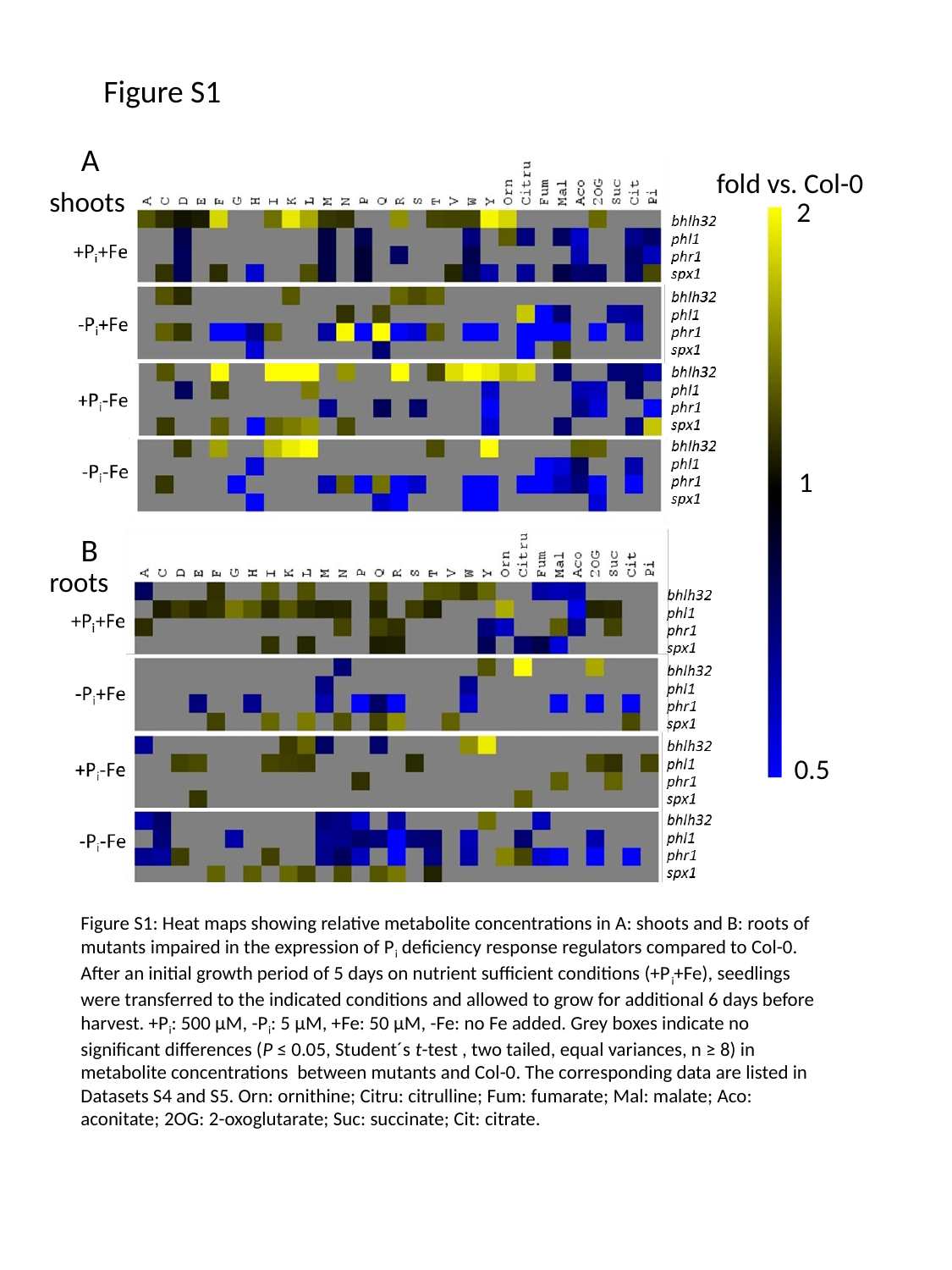

Figure S1
A
fold vs. Col-0
shoots
2
1
B
roots
0.5
Figure S1: Heat maps showing relative metabolite concentrations in A: shoots and B: roots of mutants impaired in the expression of Pi deficiency response regulators compared to Col-0. After an initial growth period of 5 days on nutrient sufficient conditions (+Pi+Fe), seedlings were transferred to the indicated conditions and allowed to grow for additional 6 days before harvest. +Pi: 500 µM, -Pi: 5 µM, +Fe: 50 µM, -Fe: no Fe added. Grey boxes indicate no significant differences (P ≤ 0.05, Student´s t-test , two tailed, equal variances, n ≥ 8) in metabolite concentrations between mutants and Col-0. The corresponding data are listed in Datasets S4 and S5. Orn: ornithine; Citru: citrulline; Fum: fumarate; Mal: malate; Aco: aconitate; 2OG: 2-oxoglutarate; Suc: succinate; Cit: citrate.
