# Supplementary material for: Modulation of Phosphate Deficiency-Induced Metabolic Changes by Iron Availability in Arabidopsis thaliana
Source: Int J Mol Sci. 2021 Jul 16;22(14):7609. doi: 10.3390/ijms22147609 (PMC8306678; doi:10.3390/ijms22147609)
Supplement: Supplementary file 1 [file ijms-22-07609-s001.zip › Chutia-etal-Table S1.pdf]

Table S1. MS parameters for MRM-transitions.

	MRM Transitions	Retention Time, Min	Ionization Energy, V	Collision Energy, V
phosphate	**315→299**	2.77	-240	20
	315→225			30
succinate	**263→173**	2.89		10
	263→247			10
[2,2,3,3-^{2}H] succinate	**267→177**			10
	267→251			10
fumarate	**261→245**	3.02		10
	261→171			10
malate	**351→233**	3.58		10
	351→189			15
[2,2,3,-^{2}H] malate	**354→236**			10
	354→192			15
2-oxoglutarate	**320→244**	3.88		5
	320→230			5
cis-aconitate	**391→211**	4.42		15
	391→301			10
citrate	**481→273**	4.66		20
	481→363			10
[2,2,4,4-^{2}H] citric acid	**485→276**			20
	485→367			10

Quantifier and qualifier transitions are indicated in bold and italics, respectively.
