# Supplementary material for: Modulation of Phosphate Deficiency-Induced Metabolic Changes by Iron Availability in Arabidopsis thaliana
Source: Int J Mol Sci. 2021 Jul 16;22(14):7609. doi: 10.3390/ijms22147609 (PMC8306678; doi:10.3390/ijms22147609)
Supplement: Supplementary file 1 [file ijms-22-07609-s001.zip › Supplemental Datafile S1/data_analysis.html]

PCA Plot of Targeted Metabolomics


# PCA Plot of Targeted Metabolomics

#### Jörg Ziegler, Sarah Scharfenberg and Steffen Neumann

#### 3/3/2020

This vignette is performint the data analysis for Arabidopsis thaliana targeted metabolomics data.

Some inspiration is taken from the 2015 class material SDS 348 by Claus O. Wilke, Department of Integrative Biology, Institute for Cellular and Molecular Biology, Austin, Texas (https://wilkelab.org/classes/SDS348/2015\_spring\_worksheets/class9.html).

First steps is to load libraryies and the data from files, and prepare the data for the tidyverse.

```
knitr::opts_chunk$set(echo = TRUE)

library(magrittr)
library(tibble)
library(tidyr)
```

```
## 
## Attaching package: 'tidyr'
```

```
## The following object is masked from 'package:magrittr':
## 
##     extract
```

```
library(plotly)
```

```
## Loading required package: ggplot2
```

```
## 
## Attaching package: 'plotly'
```

```
## The following object is masked from 'package:ggplot2':
## 
##     last_plot
```

```
## The following object is masked from 'package:stats':
## 
##     filter
```

```
## The following object is masked from 'package:graphics':
## 
##     layout
```

```
library(dplyr)
```

```
## 
## Attaching package: 'dplyr'
```

```
## The following objects are masked from 'package:stats':
## 
##     filter, lag
```

```
## The following objects are masked from 'package:base':
## 
##     intersect, setdiff, setequal, union
```

```
#setwd("/home/sneumann/data_extern/jziegler.ipb-halle.de/")

files <- c("Col0_all_treatments-roots-for_Steffen-PCA.csv",
           "Col0_all_treatments-shoots-for_Steffen-PCA.csv")
tissues <- sapply(strsplit(files, "-"), function(x) x[2])

data_list <- lapply(files, 
                    function(f) {
                      data <- read.csv(f)
                      rownames(data) <- data[,"X"]
                      data <- data[,-1]
                      
                      groups <- rep("+Pi+Fe", times=ncol(data))
                      names(groups) <- colnames(data)
                      groups[grepl(".Fe", colnames(data))] <- "+Pi-Fe"
                      groups[grepl(".P", colnames(data))] <- "-Pi+Fe"
                      groups[grepl(".P.Fe", colnames(data))] <- "-Pi-Fe"
                      
                      ## convert to tibble with variables in the columns
                      data <- as.data.frame(t(data))
                      data$Sample <- rownames(data)
                      data$Class <- groups
                      data$Tissue <- sapply(strsplit(f, "-"), function(x) x[2])
                      ## return 
                      as_tibble(data) 
                    })

names(data_list) <- tissues
metabolite_tibble <- bind_rows(data_list)
metabolite_tibble[is.na(metabolite_tibble)] <- 0.01
```

## PCA Analysis

The principle component analysis is a multivariate, unsupervised method to analyse high-dimensional data.

```
pca_list <- lapply(tissues, function(x) {
metabolite_tibble %>% filter(Tissue==x) %>% select(-Sample,-Class,-Tissue) %>% # remove Species column
  scale(center=TRUE, scale = FALSE) %>%                 # scale to 0 mean and unit variance
  prcomp() ->                 # do PCA
  pca                         # store result as `pca`
})
names(pca_list) <- tissues
```

The Scree plots help to decide how many principal components should be considered in the analysis and interpretation. After considering the first two PCs, there is a sharp drop in the additional explained variances.

```
pca_variance_list <- lapply (tissues, function(tissue) {
  as_tibble(data.frame(sdev=pca_list[[tissue]]$sdev, 
                       PC=c(1:length(pca_list[[tissue]]$sdev)), 
                       Tissue=tissue, stringsAsFactors=FALSE))
})
pca_variances <- bind_rows(pca_variance_list)

pca_variances <- pca_variances %>% 
  group_by(Tissue) %>%
  mutate(expVar = sdev/sum(sdev) )

screeplot <- ggplot(pca_variances, 
                    aes(x = PC, y = expVar)) + 
                    geom_line() + 
                    geom_hline(yintercept = 0.2) + 
                    xlab("Component Index") +
                    ylab("Variance explained") +
                    ggtitle("Scree Plot") + 
                    facet_wrap(~Tissue) + 
                    theme_light()
ggsave("screeplot-Col0.png")
ggplotly(screeplot, tooltip = "text")
```

Now the PCA results can be visualised, first the score plots for the first two PCs.

```
## Scores (rotated data is available in pca$x)
pca_data_list <- lapply (tissues, function(tissue) {
  as_tibble(data.frame(pca_list[[tissue]]$x, 
                       Sample=metabolite_tibble %>% filter(Tissue==tissue) %>% select(Sample), 
                       Class=metabolite_tibble %>% filter(Tissue==tissue) %>% select(Class),
                       Tissue=tissue, stringsAsFactors=FALSE))
})
pca_data <- bind_rows(pca_data_list)

## Concatenate Variances for axis labelling
xlabel <- paste("PC1: ", paste(100*round(unlist((pca_variances %>% filter(PC==1) %>% select("expVar"))[,"expVar"]), 3), "%", sep="", collapse="/"), sep="")
ylabel <- paste("PC2: ", paste(100*round(unlist((pca_variances %>% filter(PC==2) %>% select("expVar"))[,"expVar"]), 3), "%", sep="", collapse="/"), sep="")


scoresplot <- ggplot(pca_data, aes(x=PC1, y=PC2, color=Class, text=Sample)) + 
  geom_point() + 
  xlab(xlabel) +
  ylab(ylabel) + 
  facet_wrap(~Tissue) + 
  theme_light()

ggsave("scoresplot-Col0.png")

ggplotly(scoresplot, tooltip = "text")
```

Next the scores plot shows which metabolites contribute to the PCs.

```
## Loadings
rotation_data_list <- lapply (tissues, function(tissue) {
  as_tibble(data.frame(pca_list[[tissue]]$rotation, 
                       Metabolite=row.names(pca_list[[tissue]]$rotation),
                       Tissue=tissue, stringsAsFactors=FALSE))
})
rotation_data <- bind_rows(rotation_data_list)


rotation_data <- rotation_data %>%
  group_by(Tissue) %>%
  mutate(topPC1 = PC1 > sort(PC1,decreasing = TRUE)[3]) %>%
  mutate(topPC2 = PC2 > sort(PC2,decreasing = TRUE)[3]) %>%
  mutate(isTop = topPC1 | topPC2)

arrow_style <- arrow(length = unit(0.05, "inches"), type = "closed")

 loadingsplot <- ggplot(rotation_data, aes(x=PC1, y=PC2, text=Metabolite)) + 
  geom_point() +
  geom_text(aes(x=PC1, y=PC2, label=ifelse(isTop, Metabolite, "")), 
            position = position_nudge(x = +0, y = + 0.1),
            hjust=0, size=3, color='darkgreen') + 
  geom_segment(aes(xend=PC1*isTop, yend=PC2*isTop), x=0, y=0, arrow=arrow_style, color="grey") +
  xlab(xlabel) +
  ylab(ylabel) + 
  facet_wrap(~Tissue) + 
  theme_light()


ggsave("loadingsplot-Col0.png")
 
ggplotly(loadingsplot,tooltip = "text" )
```

## SessionInfo

This analysis was run with the following software versions.

```
sessionInfo()
```

```
## R version 3.6.2 (2019-12-12)
## Platform: x86_64-pc-linux-gnu (64-bit)
## Running under: Debian GNU/Linux 10 (buster)
## 
## Matrix products: default
## BLAS/LAPACK: /usr/lib/x86_64-linux-gnu/libopenblasp-r0.3.5.so
## 
## locale:
##  [1] LC_CTYPE=en_US.UTF-8       LC_NUMERIC=C              
##  [3] LC_TIME=en_US.UTF-8        LC_COLLATE=en_US.UTF-8    
##  [5] LC_MONETARY=en_US.UTF-8    LC_MESSAGES=C             
##  [7] LC_PAPER=en_US.UTF-8       LC_NAME=C                 
##  [9] LC_ADDRESS=C               LC_TELEPHONE=C            
## [11] LC_MEASUREMENT=en_US.UTF-8 LC_IDENTIFICATION=C       
## 
## attached base packages:
## [1] stats     graphics  grDevices utils     datasets  methods   base     
## 
## other attached packages:
## [1] dplyr_0.8.3   plotly_4.9.1  ggplot2_3.2.1 tidyr_1.0.0   tibble_2.1.3 
## [6] magrittr_1.5 
## 
## loaded via a namespace (and not attached):
##  [1] Rcpp_1.0.3        later_1.0.0       pillar_1.4.3      compiler_3.6.2   
##  [5] tools_3.6.2       zeallot_0.1.0     digest_0.6.23     jsonlite_1.6     
##  [9] evaluate_0.14     lifecycle_0.1.0   gtable_0.3.0      viridisLite_0.3.0
## [13] pkgconfig_2.0.3   rlang_0.4.2       shiny_1.4.0       crosstalk_1.0.0  
## [17] yaml_2.2.0        xfun_0.11         fastmap_1.0.1     withr_2.1.2      
## [21] stringr_1.4.0     httr_1.4.1        knitr_1.26        vctrs_0.2.1      
## [25] htmlwidgets_1.5.1 grid_3.6.2        tidyselect_0.2.5  glue_1.3.1       
## [29] data.table_1.12.8 R6_2.4.1          rmarkdown_2.0     farver_2.0.1     
## [33] purrr_0.3.3       promises_1.1.0    ellipsis_0.3.0    backports_1.1.5  
## [37] scales_1.1.0      htmltools_0.4.0   assertthat_0.2.1  xtable_1.8-4     
## [41] mime_0.8          colorspace_1.4-1  httpuv_1.5.2      labeling_0.3     
## [45] stringi_1.4.3     lazyeval_0.2.2    munsell_0.5.0     crayon_1.3.4
```
