# Supplementary figures and images for: Modulation of Phosphate Deficiency-Induced Metabolic Changes by Iron Availability in Arabidopsis thaliana
Source: Int J Mol Sci. 2021 Jul 16;22(14):7609. doi: 10.3390/ijms22147609 (PMC8306678; doi:10.3390/ijms22147609)

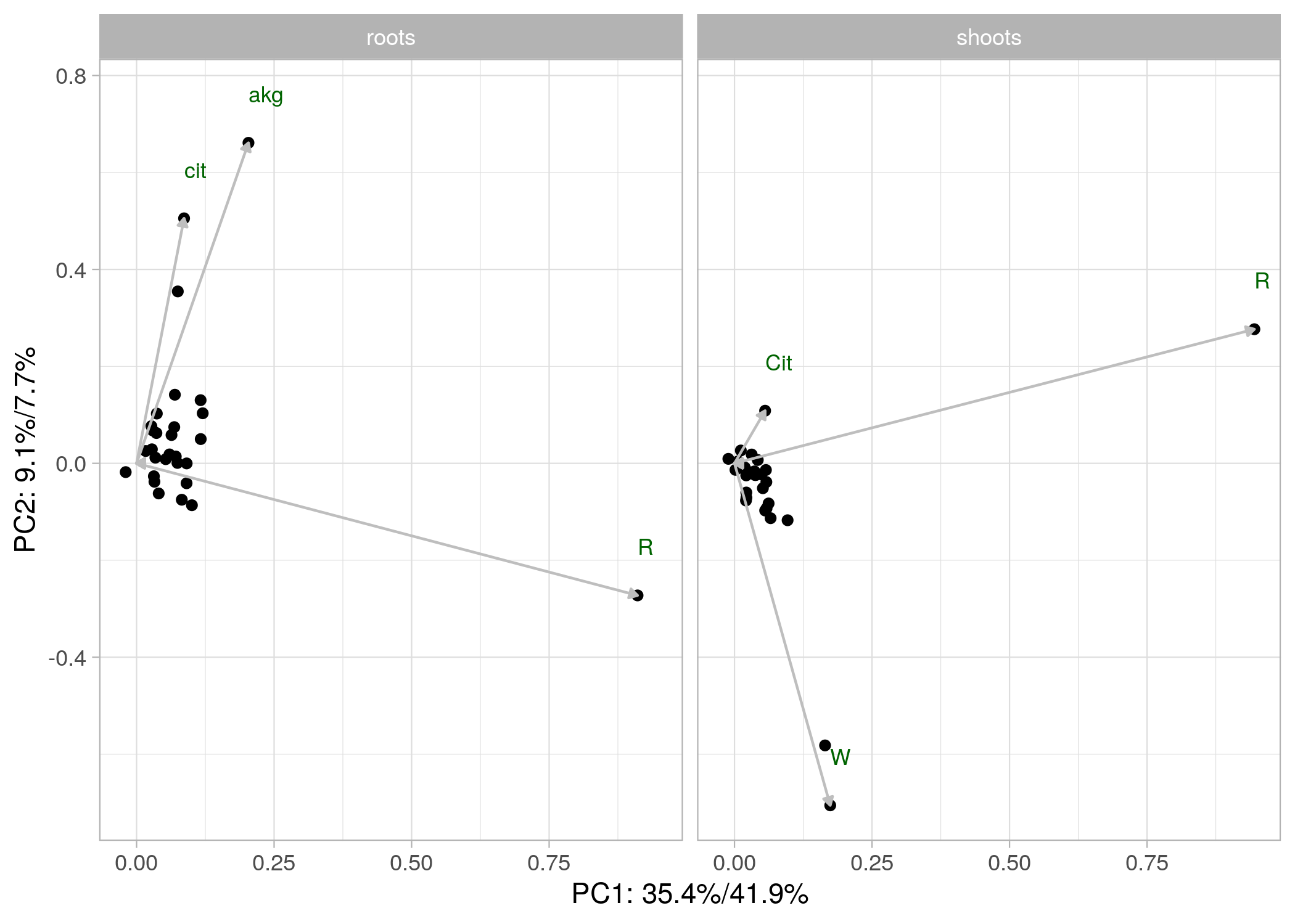

Supplement: Supplementary file 1 [file ijms-22-07609-s001.zip › Supplemental Datafile S1/loadingsplot-Col0.png]

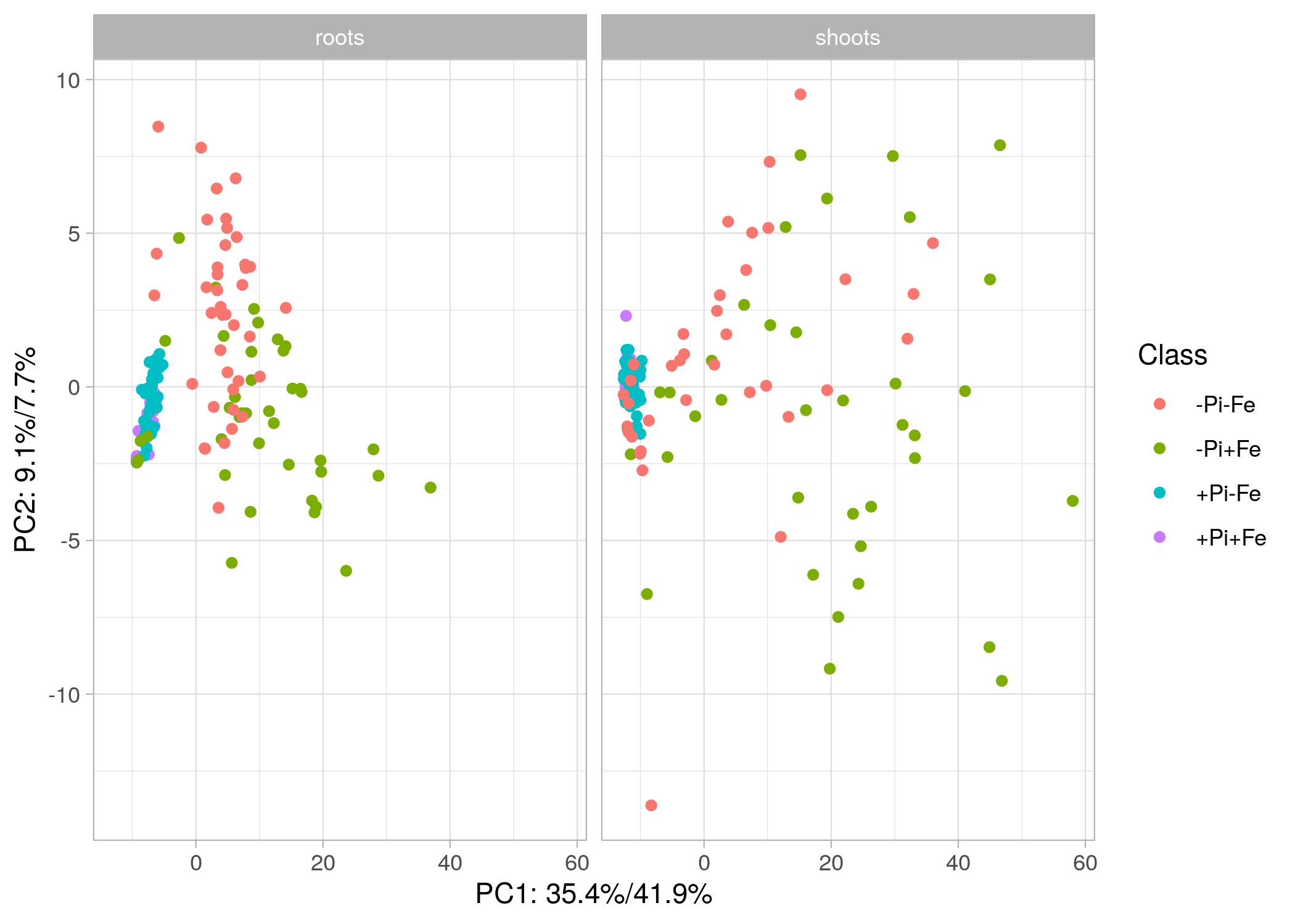

Supplement: Supplementary file 1 [file ijms-22-07609-s001.zip › Supplemental Datafile S1/scoresplot-Col0.png]

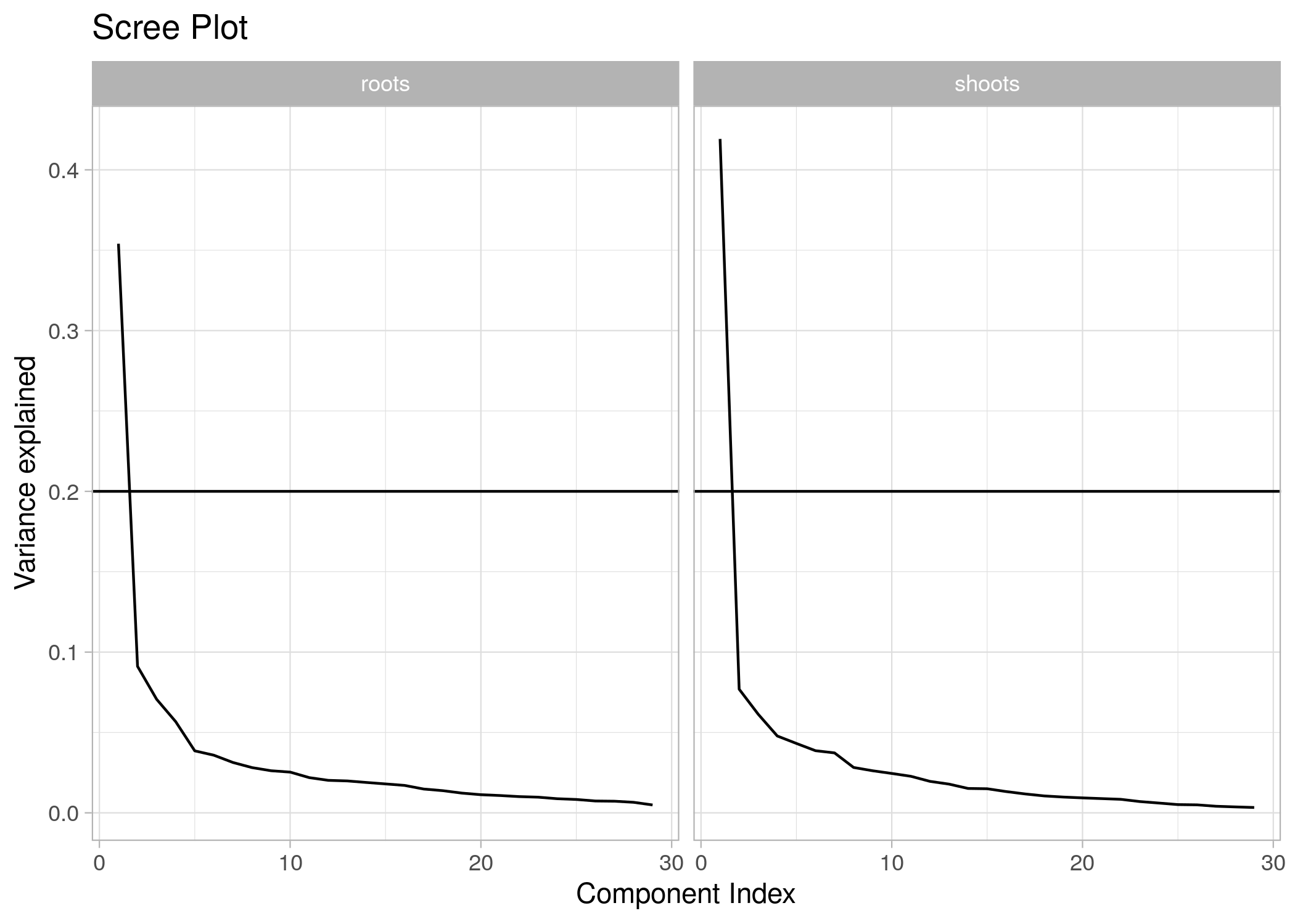

Supplement: Supplementary file 1 [file ijms-22-07609-s001.zip › Supplemental Datafile S1/screeplot-Col0.png]
